# Supplementary figures and images for: Dbx2, an Aging-Related Homeobox Gene, Inhibits the Proliferation of Adult Neural Progenitors
Source: Stem Cell Rev Rep. 2023 Aug 22;19(8):2837–51. doi: 10.1007/s12015-023-10600-7 (PMC10661760; doi:10.1007/s12015-023-10600-7)

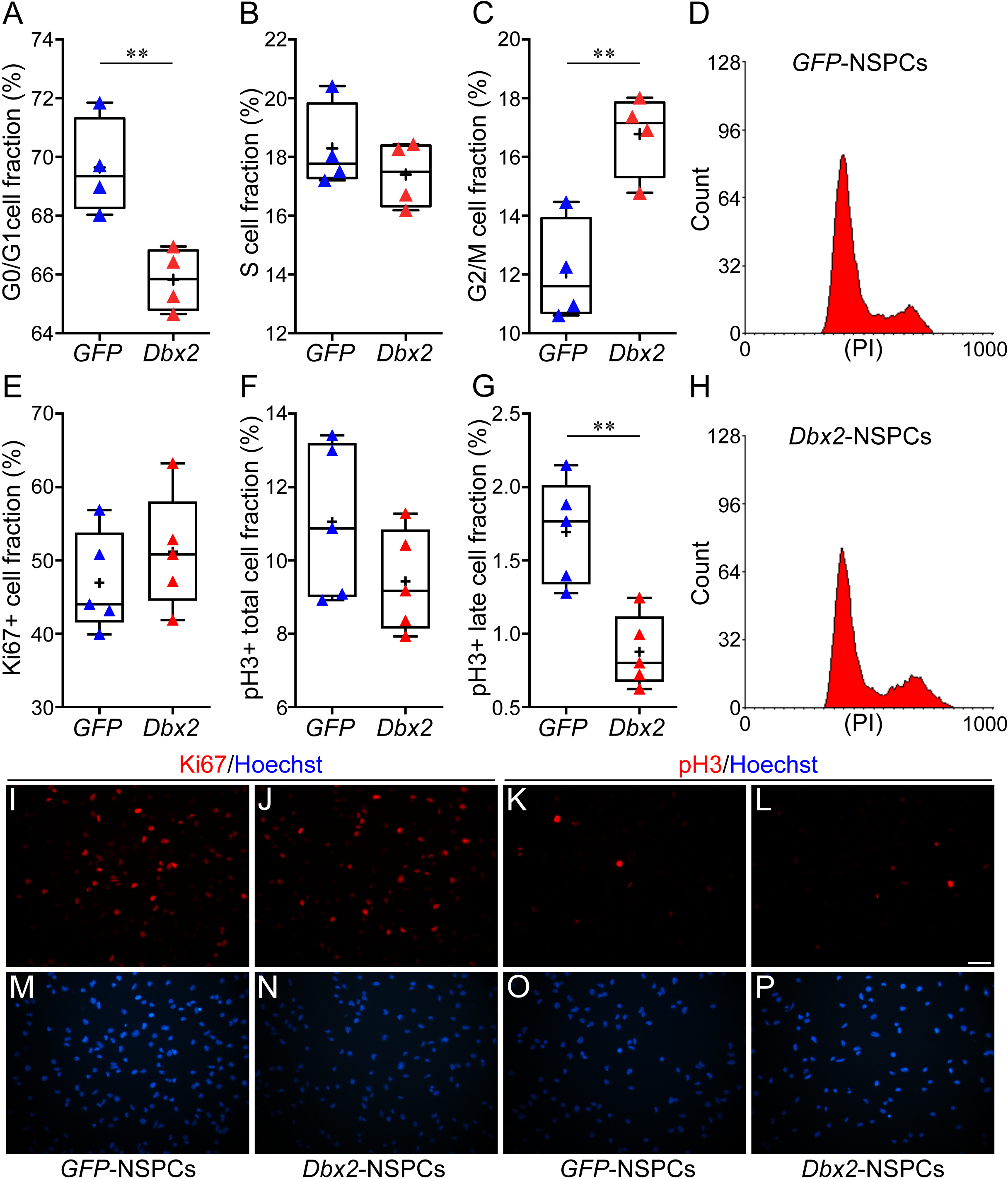

Supplement: Supplementary file 1 — Figure S1. Validation of the effects of constitutive Dbx2 overexpression in an independent pair of GFP-NSPCs and Dbx2-NSPCs. (A to C) Box-and-whisker plots of the fraction of GFP-NSPCs (blue triangles) and Dbx2-NSPCs (red triangles) in the G0/G1 (A), S (B) and G2/M (C) phases of the cell cycle, after culture of an independent pair of transgenic NSPCs (n=4); **, p < 0.01, Student’s t-test. Flow cytometry histograms of PI-stained GFP-NSPC and Dbx2-NSPC cultures from a representative experiment are shown in (D) and (H), respectively. (E to G) Box-and-whisker plots of the fraction of Ki67+ (E), pH3+ total (F) and pH3+ late (G) cells, after culture of an independent pair of GFP-NSPCs (blue triangles) and Dbx2-NSPCs (red triangles) (n=5); **, p < 0.01, Student’s t-test. (I to P) Representative images of GFP-NSPC (I, M, K, O) and Dbx2-NSPC (J, N, L, P) cultures stained with anti-Ki67 (I, J) or anti-pH3 (K, L) antibodies. Hoechst nuclear staining is shown in (M to P). Scale bar, 40 μm. (PNG 2100 kb) [file 12015_2023_10600_Fig7_ESM.png]

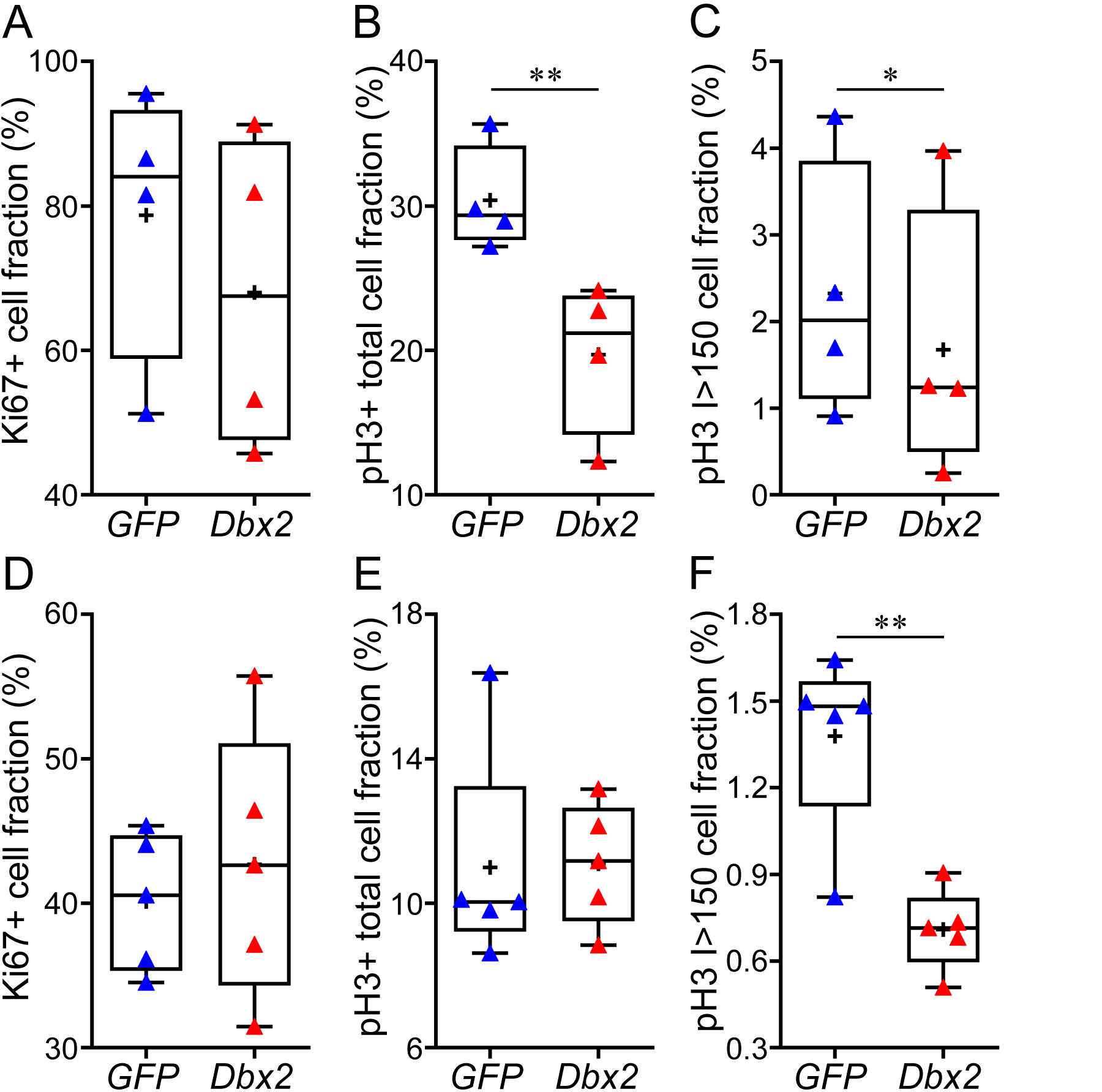

Supplement: Supplementary file 3 — Figure S2. Validation of the effects of constitutive Dbx2 overexpression by automated immunofluorescence analysis. (A to F) Box-and-whisker plots of the fraction of Ki67+ cells (A, D), pH3+ total cells (B, E), and pH3+ cells with fluorescent intensity above 150 units/pixel (C, F), after culture of two independent pairs of GFP-NSPCs (blue triangles) and Dbx2-NSPCs (red triangles), and automated immunofluorescence analysis using ImageJ. (A to C) Results obtained with the same GFP-NSPC and Dbx2-NSPC lines as shown in Fig. 2 (n=4); (D to F) Results obtained with the same GFP-NSPC and Dbx2-NSPC lines as shown in Fig. S1 (n=5); *, p < 0.05, **, p < 0.01, Student’s t-test. (PNG 135 kb) [file 12015_2023_10600_Fig8_ESM.png]

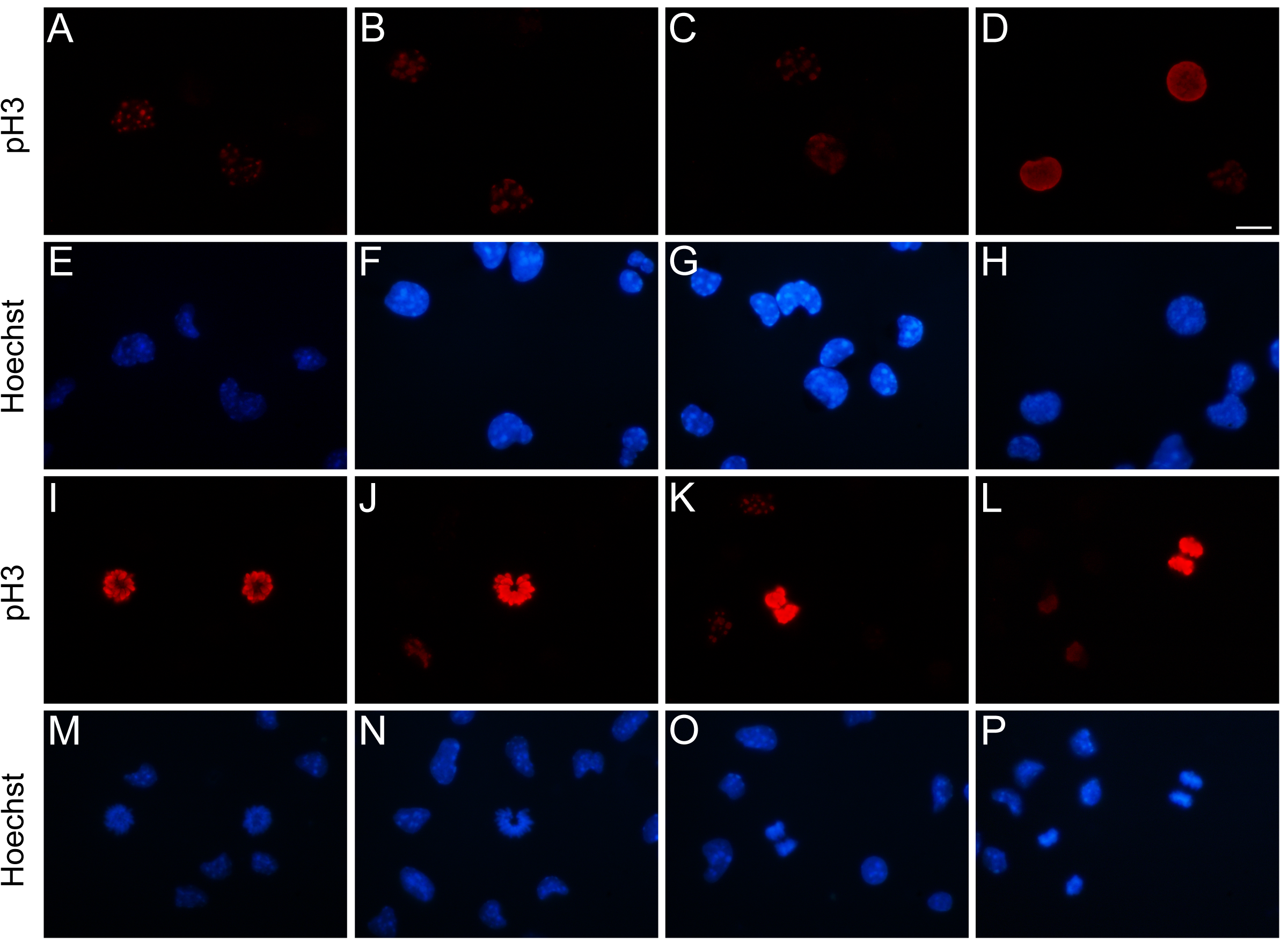

Supplement: Supplementary file 5 — Figure S3. Dynamic pattern of nuclear pH3 localization in young adult NSPCs going through the G2/M transition. (A to D and I to L) Representative high magnification images of young adult NSPCs stained with an anti-pH3 antibody, showing the pattern of nuclear pH3 staining during late G2 to early prophase (A to C), late prophase to metaphase (D, I, J), anaphase to telophase (K, L). Hoechst nuclear staining is shown in (E to H) and (M to P). Scale bar, 10 μm. (PNG 3871 kb) [file 12015_2023_10600_Fig9_ESM.png]

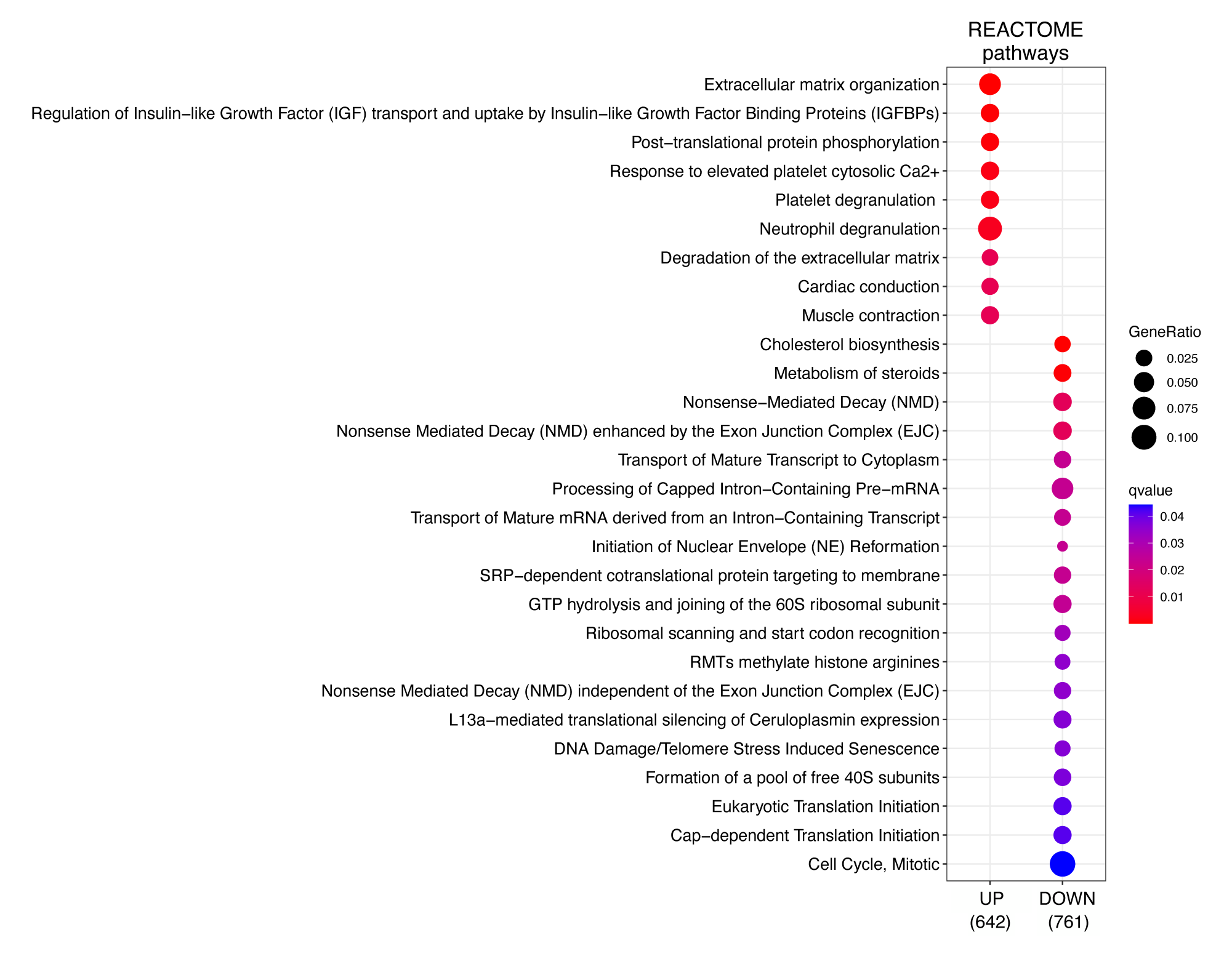

Supplement: Supplementary file 7 — Figure S4. REACTOME pathway enrichment in Dbx2-modulated genes. Dot plot showing the REACTOME pathways that are enriched in the upregulated (left) and the downregulated (right) DEGs in Dbx2-NSPCs vs GFP-NSPCs. The size of the dots is based on the count of the DEGs correlated with each pathway; the colour of the dots shows the qvalue associated with each pathway. (PNG 159 kb) [file 12015_2023_10600_Fig10_ESM.png]

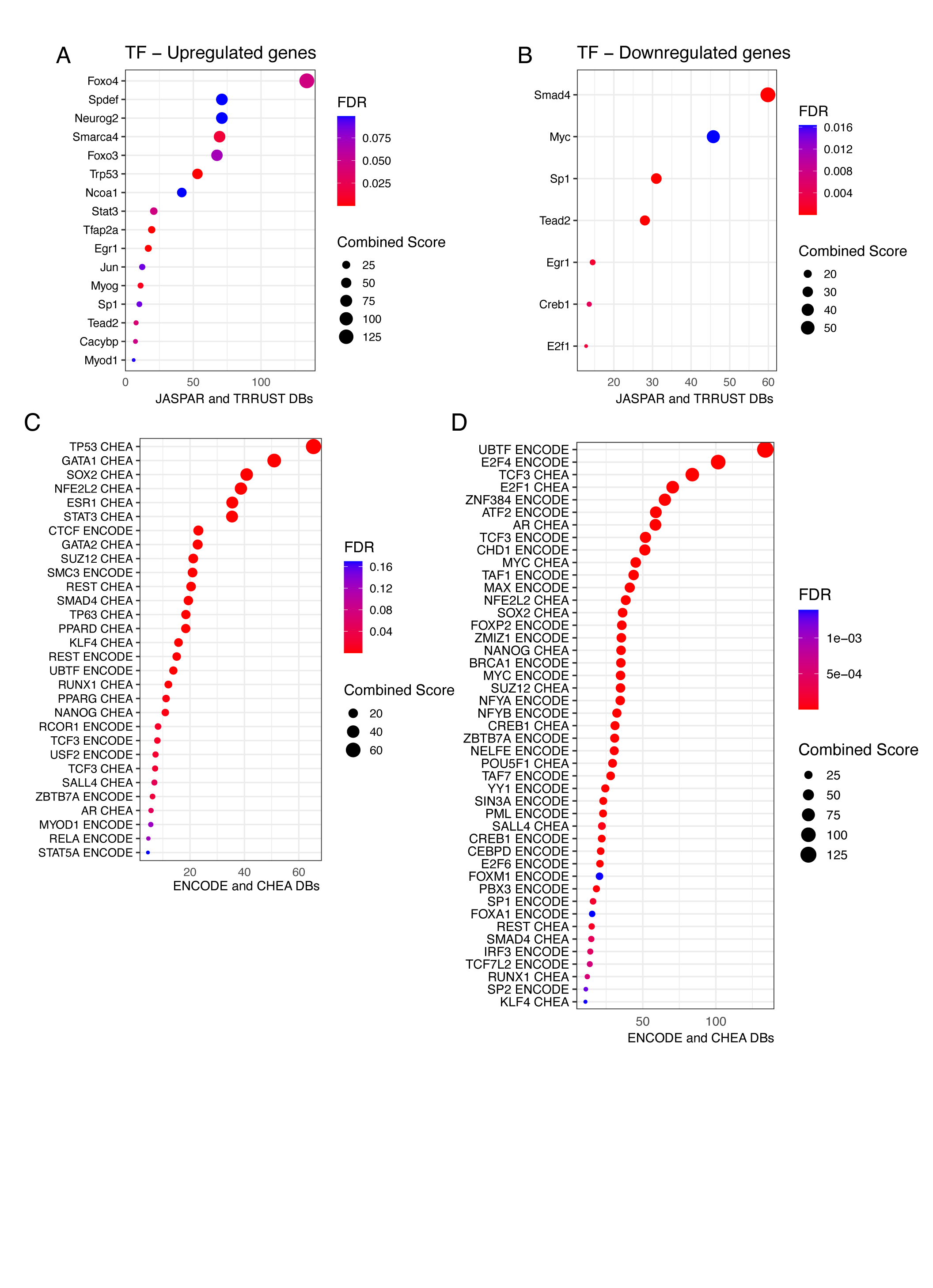

Supplement: Supplementary file 9 — Figure S5. Transcription factor motifs enriched in Dbx2-modulated genes. (A to D) Dot plots showing the transcription factor motifs enriched in the upregulated (A, C) and the downregulated (B, D) DEGs in Dbx2-NSPCs vs GFP-NSPCs, according to the JASPAR and TRRUST databases (A, B) or the ENCODE and CHEA databases (C, D). The size of the dots is based on the combined score associated with each motif, as defined by Enrichr method [48]; the colour of the dots shows the FDR value associated with each motif. (PNG 310 kb) [file 12015_2023_10600_Fig11_ESM.png]

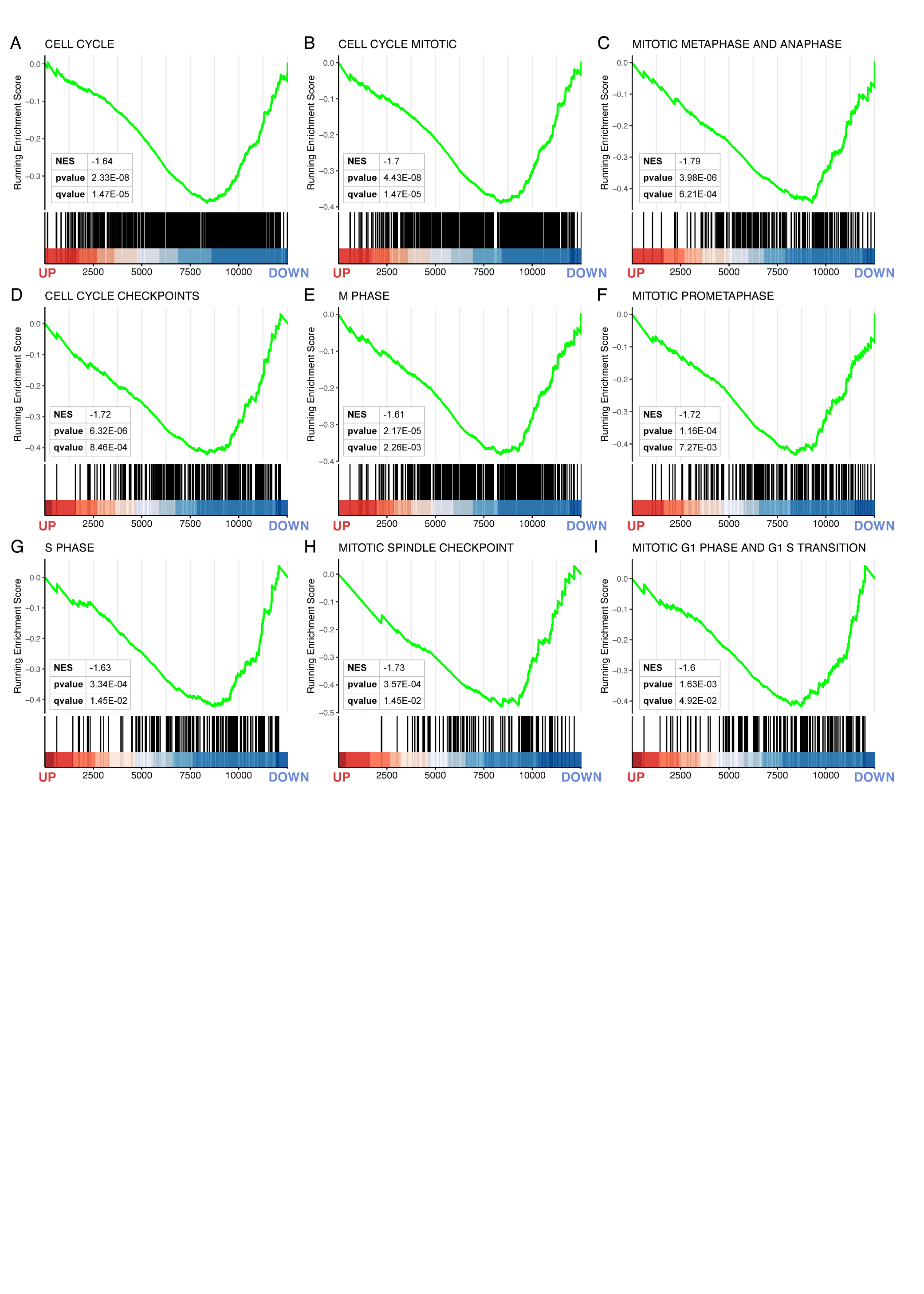

Supplement: Supplementary file 11 — Figure S6. Dbx2 overexpression inhibits the expression of gene sets associated with different cell cycle phases. (A to I) Enrichment plots showing the enrichment in the Dbx2-associated signature of gene sets related to different cell cycle phases, which were obtained from the “C2” curated gene sets collection in MSigDB, according to GSEA. Vertical black lines indicate individual members of each gene set and their position within the ranked Dbx2-associated signature; the heat maps at the bottom of the plots highlight the proportion of Dbx2-downregulated genes (blue colour) and Dbx2-upregulated genes (red colour) in each gene set. (PNG 227 kb) [file 12015_2023_10600_Fig12_ESM.png]

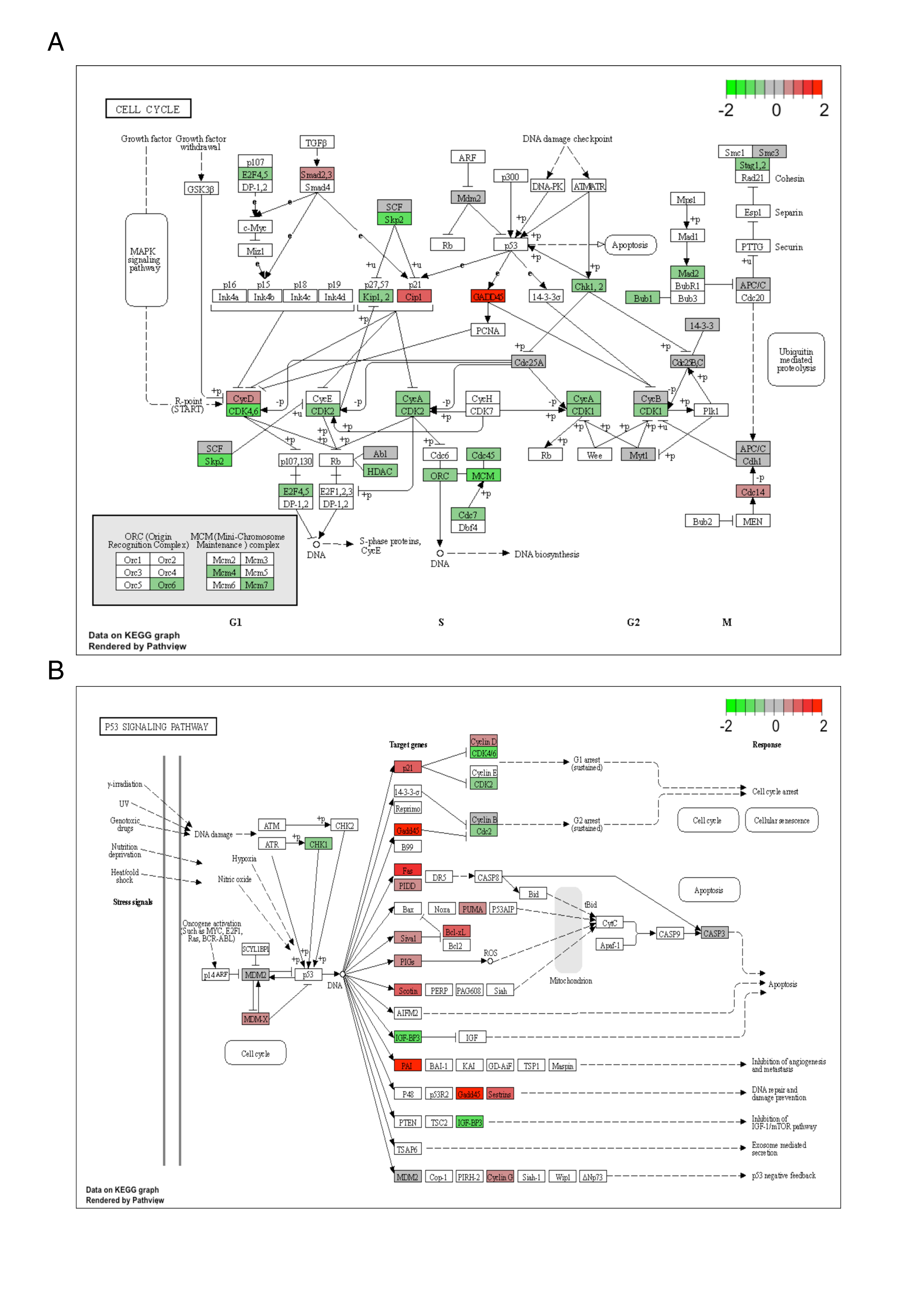

Supplement: Supplementary file 13 — Figure S7. Dbx2 overexpression causes the downregulation of genes involved in cell cycle progression and the upregulation of genes involved in cell cycle inhibition. (A, B) Pathview-generated diagrams of the cell cycle molecular pathway (A), or the p53 signaling pathway (B), according to KEGG database, showing that genes with crucial roles in cell cycle progression are downregulated in Dbx2-overexpressing NSPCs (green colour, FC < 0), whereas genes coding for key cell cycle inhibitors are upregulated following Dbx2 overexpression (red colour, FC > 0). (PNG 818 kb) [file 12015_2023_10600_Fig13_ESM.png]
